# Creation of a novel peptide with enhanced nuclear localization in prostate and pancreatic cancer cell lines

**DOI:** 10.1186/1472-6750-10-79

**Published:** 2010-10-28

**Authors:** H Dan Lewis, Ali Husain, Robert J Donnelly, Dimitrios Barlos, Sheraz Riaz, Kalyani Ginjupalli, Adetola Shodeinde, Beverly E Barton

**Affiliations:** 1 Department of Surgery, University of Medicine and Dentistry of New Jersey, New Jersey Medical School, 185 S. Orange Avenue, Newark, NJ 07103 USA; 2 Molecular Resources Facility, University of Medicine and Dentistry of New Jersey, New Jersey Medical School, USA; 3 Department of Pharmacology & Physiology, University of Medicine and Dentistry of New Jersey, New Jersey Medical School, USA; 4 Department of Veterans Affairs - New Jersey Health Care System, 385 Tremont Avenue, East Orange, NJ 07018 USA; 5 Current Address: VaxInnate, 3 Cedarbrook Drive, Cranbury, NJ 08512 USA

## Abstract

**Background:**

For improved uptake of oligonucleotide-based therapy, the oligonucleotides often are coupled to peptides that facilitate entry into cells. To this end, novel cell-penetrating peptides (CPPs) were designed for mediating intracellular uptake of oligonucleotide-based therapeutics. The novel peptides were based on taking advantage of the nuclear localization properties of transcription factors in combination with a peptide that would bind putatively to cell surfaces. It was observed that adding a glutamate peptide to the N-terminus of the nuclear localization signal (NLS) of the Oct6 transcription factor resulted in a novel CPP with better uptake and better nuclear colocalization than any other peptide tested.

**Results:**

Uptake of the novel peptide Glu-Oct6 by cancer cell lines was rapid (in less than 1 hr, more than 60% of DU-145 cells were positive for FITC), complete (by 4 hr, 99% of cells were positive for FITC), concentration-dependent, temperature-dependent, and inhibited by sodium azide (NaN_3_). Substitution of Phe, Tyr, or Asn moieties for the glutamate portion of the novel peptide resulted in abrogation of novel CPP uptake; however none of the substituted peptides inhibited uptake of the novel CPP when coincubated with cells. Live-cell imaging and analysis by imaging flow cytometry revealed that the novel CPP accumulated in nuclei. Finally, the novel CPP was coupled to a carboxyfluorescein-labeled synthetic oligonucleotide, to see if the peptide could ferry a therapeutic payload into cells.

**Conclusions:**

These studies document the creation of a novel CPP consisting of a glutamate peptide coupled to the N-terminus of the Oct6 NLS; the novel CPP exhibited nuclear colocalization as well as uptake by prostate and pancreatic cancer cell lines.

## Background

Experimental therapeutic approaches using oligonucleotides for prostate and pancreatic cancer are actively investigated in many laboratories, including ours [1,2]. Such inhibitors are attractive in theory but lack a practical method for delivery in the clinical setting. One possible approach to overcome this roadblock is to use peptide-mediated transport, thereby coupling a cell-penetrating peptide (CPP) to a therapeutic payload, such as a peptide nucleic acid (PNA). An inherent advantage of using CPPs is the ability to design cell specificity in the sequence, as well as target organelle specificity through inclusion of nuclear localization signals (NLS). CPP-mediated can be quite efficient, allowing for rapid and complete uptake and delivery of a PNA payload for the treatment of HIV [3].

CPPs for delivery of therapeutic oligonucleotides have gained attention in recent years; an excellent review describing the major categories of CPPs was published earlier this year [4]. CPPs for prostate cancer have been examined in conjunction with delivery of methotrexate-loaded liposomes [5,6], double-stranded decoys [7], and radioactive gadolinium complexes targeted to *c-myc *[8]. As for pancreatic cancer, the antennepedia protein Antp when coupled to the tumor suppressor p16 successfully inhibited cell growth [9], and the insulin-like growth factor loop 1 peptide IGF1 is being tried for imaging of early pancreatic tumors [10].

Our laboratory has been involved in STAT3 inhibition for cancer therapy for a number of years. Previously, we designed oligonucleotides that inhibited STAT3 expression with concomitant abrogation of STAT3 target gene expression [1,2]. It had long been our intention to use PNAs as therapeutic entities for STAT3, given the superior properties of PNAs compared to oligonucleotides for this purpose. PNAs bind strongly to RNA or DNA, more strongly than antisense or RNAi, thereby inhibiting transcription of gene(s) through the creation of triple helices. The structure of PNAs makes them highly resistant to nucleases and proteases [11]. Finally, PNAs form triple helices with duplex DNA, making them ideal candidate molecules for inhibiting transcription factors [12]. However, PNAs need suitable CPPS for transport into cells. And in the case of inhibiting a transcription factor such as STAT3, nuclear colocalization is highy desirable since the nucleus is the main seat of transcription factor activity.

One strategy for CPP design recently examined is to use the NLS peptides of transcription factors themselves as CPPs. The NLS of several transcription factors have been compared in various tumor types with varying degrees of efficacy with regard to uptake and nuclear localization, however sequestration in endosomes was observed for many of the peptides tested [13]. As for delivery of an oligonucleotide or PNA payload, one study using CPPs consisting of cell surface ligands linked to NLS and conjugated to peptide nucleic acids (PNAs) found optimal efficacy under serum-free conditions at 5 mM, a concentration that is not commercially feasible due to prohibitive costs [14]. Clearly, more studies on designing and optimizing CPPs for delivery of therapeutic oligonucleotide or PNA payloads are needed in order to bring new therapeutic entities to the clinic.

In this paper, the creation of a novel CPP combining the NLS of a transcription factor with another peptide that was observed to enhance cellular uptake and nuclear localization. By adding a glutamate-rich peptide (EEEAA) to the N-terminus of the Oct6 transcription factor NLS, a novel CPP was created that entered prostatic and pancreatic cancer cell lines readily. As little as 30 nM of the FITC-labeled glutamate-rich peptide was sufficient to stain 60-70% of the cells. Addition of a lysyl peptide, KKK, to the glutamate-rich peptide enhanced uptake 10-fold, and addition of the Oct6 NLS to the glutamate-rich peptide enhanced uptake 100-fold. Combining the glutamate peptide to the Oct6 NLS created a novel peptide with better nuclear localization properties than either the glutamate-rich peptide or the Oct6 NLS alone. Substituting other amino acids for the glutamate residues abrogated uptake, but coincubation of substituted peptides with the novel CPP did not inhibit uptake, meaning that uptake of the novel CPP was sequence-specific. Finally, when the novel peptide was coupled to a carboxyfluorescein-labeled PNA, uptake and nuclear localization by DU-145 cells was observed. The rational design of CPPs using the NLS of transcription factors for enhanced cancer cell uptake is worthy of study for delivery of therapeutic payloads into targeted cells.

## Results

### Neither a PMSA-targeted peptide nor TAT resulted in efficient PNA uptake in DU-145 cells

**Figure 1:**
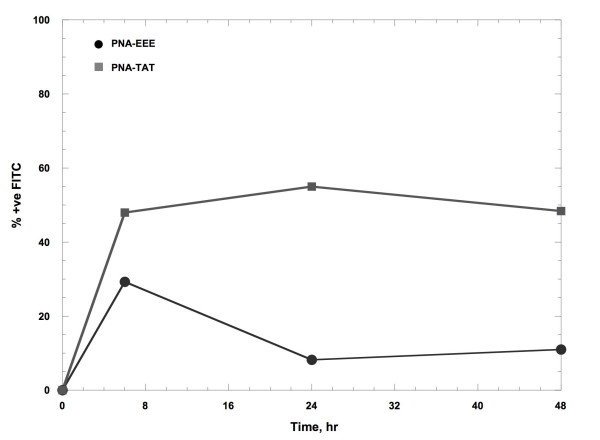
**Glu peptide or TAT was an inefficient CPP for PNA uptake in DU-145 cells**. DU-145 cells were incubated with 1000 nM FITC-PNA-EEE or FITC-PNA-TAT; at the indicated times cells were harvested and analyzed by flow cytometry for FITC fluorescence.

Because PMSA (prostate-specific membrane antigen) is overexpressed on most prostate cancer cells, it is an attractive target for therapeutic delivery. Being a carboxyglutamate peptidase, it cleaves C-terminal Glu residues. C-terminal peptides such as EEE have been used previously to deliver methotrexate-loaded liposomes to prostate cancer cell lines [6]; whether a C-terminal Glu peptide could deliver a PNA to prostate cancer cells had not been investigated. TAT peptide, a 13-amino acid peptide comprised of residues 48 to 60 of the human immunodeficiency virus-1 TAT protein, has been shown to be an efficient CPP for delivery of anti-viral oligonucleotides and PNAs [3]; as is the case for EEE, it had not been fully evaluated in most cancer lines for delivery of PNAs. Therefore, FITC-labeled PNAs bearing either a carboxy-terminal peptide (EEE) or an N-terminal TAT peptide were synthesized and incubated with DU-145 cells (see Table 1 for sequences of all peptides and peptide-PNAs used). Surprisingly, only about 30-50% of the DU-145 cells were fluorescent 6 hr after start of incubation (Figure 1) and the proportion of fluorescent cells markedly decreased over time in the case of the EEE-labeled PNA (in the case of the TAT-labeled PNA, the proportion of fluorescent cells remained nearly constant over 48 hr). Based on these results, it was decided to try other strategies for design of suitable CPPs to deliver PNAs into cancer cell lines.

**Table 1:** Peptides and Peptide-PNAs Included in Studies

Description	Sequence	Molecular Weight
Glu-FITC	EEE-FITC	776 Da
Glu-Ala-FITC	EEEAA-FITC	919 Da
Glu-Lys-FITC	EEEAAKKK-FITC	1303 Da
Glu-Oct6-FITC	EEEAAGRKRKKRT-FITC	1930 Da
Oct6- FITC	GRKRKKRT-FITC	1400 Da
Phe-Oct6-FITC	FFFAAGRKRKKRT-FITC	1984 Da
Asn-Oct6-FITC	NNNAAGRKRKKRT-FITC	1885 Da
Phe-Oct6-dansyl	FFFAAGRKRKKRT-dansyl	1875 Da
Asn-Oct6-dansyl	NNNAAGRKRKKRT-dansyl	2058 Da
Glu-Oct6	EEEAAGRKRKKRT	1559 Da
Phe-Oct6	FFFAAGRKRKKRT	1613 Da
Tyr-Oct6	YYYAAGRKRKKRT	1661 Da
Tyr-Oct6-FITC	YYYAAGRKRKKRT	2032 Da

**Description**	**Sequence**	

FITC-PNA-EEE	FITC-**TATGATCTCCTCCGT**-EEE	
FITC-TAT-PNA	FITC-GRKKRRQRRRRC-**TATGATCTCCTCCGT**	
Glu-Oct6-PNA-FITC	EEEAAGRKRKKRT-**TATGATCTCCTCCGT**-K-FITC	
Glu-Oct6-13410a	EEEAAGRKRKKRT-**TCCCGTAAATCCCTA**	
13410a	**TCCCGTAAATCCCTA**	

Carboxyfluorescein and carboxydansyl peptides were synthesized by *FMoc *chemistry at the Molecular Resources Facility, UMDNJ. They were purified by HPLC and sequence verified by the same technique. Carboxydansyl peptides are synthesized using lysyl dansyl moieties. The peptide-PNAs were synthesized by BioSynthesis; they were purified by HPLC and verified by MALDI-TOF. The letters in **bold **indicate the PNA portions of the molecules. The FITC moiety was coupled to Glu-Oct6-PNA as a lysyl-FITC, due to the requirements of PNA chemistry.

### Addition of glutamate-rich peptide EEEAA to the Oct6 NLS enhanced its uptake

**Figure 2:**
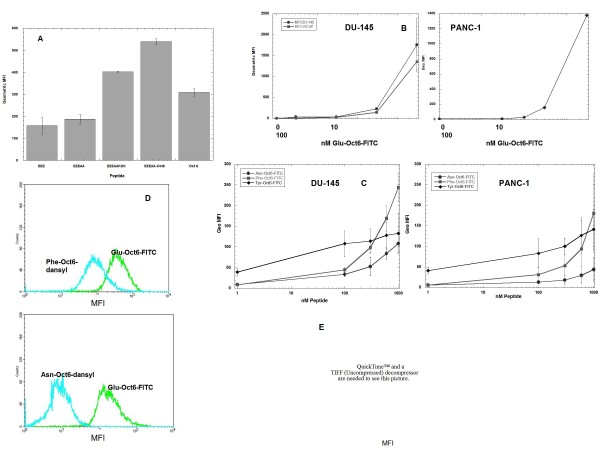
**Peptide uptake by prostate cancer cell lines**. **Figure 2A: **Enhancement of Oct6 NLS peptide uptake by addition of Glu-Ala peptide EEEAA on the N terminus. DU-145 cells were incubated with 500 nM peptides for 4 hours in Materials and Methods. Harvested washed cells were subjected to flow cytometry on a FACScan. Dead cells were excluded by gating on 7-AAD-negative cells. * = geometric MFI of Glu-Lys-FITC or Glu-Oct6-FITC was significantly greater than Glu-FITC (p < 0.01) or 356 (p < 0.05) by paired ANOVA. The average of 3 replicate experiments is shown. **Figure 2B: **Effect of concentration on uptake of Glu-Oct6-FITC by DU-145, LNCaP and PANC-1 cells. Concentrations of Glu-Oct6-FITC ranging from 0 to 300 nM were incubated with cells for 1 hr, at which time cells were harvested, washed, and processed for flow cytometry. **Figure 2C: **Effect of substituting Phe, Asn, or Tyr for Glu on Oct6 NLS peptide uptake by DU-145 and PANC-1 cells. Cells were incubated with 0 to 1000 nM of peptides for 1 hr at 37°C, then were harvested, washed, counterstained, and analyzed by flow cytometry to quantify fluorescence. The average of 3 independent experiments ± SD is shown. **Figure 2D: **Lack of competition by Phe-Oct6-Dansyl or Asn-Oct6-Dansyl on Glu-Oct6-FITC uptake by DU-145 cells. 300 nM of either Phe-Oct6-Dansyl or Asn-Oct6-Dansyl were incubated with 300 nM Glu-Oct6-FITC for 1 hr, at which time cells were harvested, washed, and analyzed on a BD LSR II for dansyl and FITC fluorescence. CellQuest Pro was used to analyze the fluorescence data. Turquoise line indicates dansyl fluorescence intensity; green line indicates fluorescein fluorescence intensity. **Figure 2E: **Effect of 250-1000 nM Phe-Oct6 on 500 nM Glu-Oct6-FITC. DU-145 cells were incubated with peptides as before for 4 hrs; cells were harvested, washed, fixed, then analyzed for fluorescence by flow cytometry. Green = no peptide; pink = Glu-Oct6-FITC alone; blue = Glu-Oct6-FITC + 250 nM Phe-Oct6; red = Glu-Oct6-FITC + 500 nM Phe-Oct6; purple = Glu-Oct6-FITC + 1000 nM Phe-Oct6.

Uptake of peptides by live cells was compared by flow cytometry, using either 7-amino actinomycin D (7-AAD) or propidium iodide (PI) to exclude dead cells from analyses. Because logarithmic amplification of fluorescence detectors were employed, the geometric mean fluorescence intensities (MFIs) were compared among samples. In comparing the geometric MFIs among the peptides, it was noted that peptides Glu-FITC and Glu-Ala-FITC (see Table 1 for sequences) exhibited the lowest values, whereas addition of either KKK or the Oct6 NLS (peptides Glu-Lys-FITC or Glu-Oct6-FITC) significantly increased the geometric MFIs after 4 hrs incubation. These data suggested the possibility that inclusion of even a minimal NLS such as KKK could profoundly influence cellular uptake. The influence of the Oct6 NLS peptide on uptake was even more dramatic; however, uptake of the Oct6 NLS alone (that is, peptide Oct6-FITC) was not nearly as great as that of Glu-Oct6-FITC. Therefore it is possible that both parts of the Glu-Oct6 peptide, EEEAA and the Oct6 NLS, contributed to the enhanced property of Glu-Oct6-FITC to function as a CPP. The kinetics of uptake for Glu-Oct6-FITC were found to be rapid, although saturation was not reached. Figure 2B shows that in both DU-145 and LNCaP cells, the geometric MFI of 500 nM at 1 hr extrapolated from the graph is nearly the same as that measured at 4 hr (Figure 2A). In the presence of 3 mM, the geometric MFIs of the two cell lines was 1500 to 2000, and uptake was still linear. Inclusion of higher concentrations of peptide was not feasible, due to the limited amount of peptide.

### Substitution of Phe, Asn, or Tyr for Glu abrogated peptide uptake

In order to determine if uptake of peptide Glu-Oct6-FITC was sequence-specific, Glu residues were substituted by Phe residues (peptides Phe-Oct6-FITC, Phe-Oct6-Dansyl, and Phe-Oct6), Asn residues (peptide Asn-Oct6-FITC), or Tyr residues (peptides Tyr-Oct6-FITC and Tyr-Oct6). The resulting peptides were incubated at 0 to 1000 nM with cells for 1 hr. Cells were then harvested and processed for analysis by flow cytometry. Figure 2C shows that there was very little uptake of peptides Phe-Oct6-FITC, Asn-Oct6-FITC, or Tyr-Oct6-FITC up to 300 nM (geometric MFI increased from approximately 40 to 100; the increase was not significant by paired ANOVA). Coincubation of GLu-Oct6-FITC with either carboxydansyl peptides Phe-Oct-Dansyl or Asn-Oct-Dansyl resulted in nearly unaltered uptake of peptide Glu-Oct6-FITC without concomitant uptake of either Phe-Oct6-Dansyl or Asn-Oct6-Dansyl (Figure 2D). Because labeling the Phe-Oct6 peptide with either FITC or dansyl resulted in peptides that had cytotoxicity at high concentrations (300 and 1000 nM; data not shown), the experiment was performed again using unlabeled Phe-Oct6 peptide in the presence of FITC-labeled Glu-Oct6-FITC, to see if Phe-Oct6 could compete with Glu-Oct6-FITC for binding and uptake. The results shown in Figure 2E reveal that incubating cells with Phe-Oct6 in the presence of Glu-Oct6-FITC did not affect uptake of Glu-Oct6-FITC. All these data taken together demonstrate that substituting Asn, Phe, or Tyr for Glu did not interfere with uptake of Glu-Oct6-FITC, indicating that uptake of Glu-Oct6-FITC had some specificity requirement for Glu on the peptide.

To check for cytotoxicity in the unlabeled Phe-Oct6 and Glu-Oct6 peptides, these peptides were incubated with PANC-1 and DU-145 cells for 18 and 48 hrs. At the appropriate times, cells were harvested and stained with FITC-labeled annexin V plus PI and analyzed by flow cytometry, in order to assess induction of apoptosis. Cell line viabilities at the start of the experiments was 98% (determined by fluorescein diacetate staining and microscopy); after treatment with Phe-Oct6 or Glu-Oct6 peptides, cell viabilities were observed to be 97 to 100% (Table 2), indicating that the cytotoxicities seen with the FITC- or dansyl-labeled Phe-Oct6 peptides were not due to the peptide part of those molecules, but rather due to the combination of the peptide plus the fluorochromes (perhaps due to the high hydrophobicity of those particular peptides). The conclusion from this series of experiments is that Glu-Oct6 and Phe-Oct6 peptides in and of themselves are not cytotoxic and do not induce apoptosis. The results of the experiments presented in this subsection are evidence that uptake is specific to the sequence of the Glu-Oct6 peptide.

**Table 2:** Cell Viability after Incubation with Peptides Glu-Oct6 and Phe-Oct6

Cell Line	Peptide	Concentration, nM	% Viability
DU-145	Glu-Oct6	0	100
		300	100
		1000	100
	Phe-Oct6	0	100
		300	99
		1000	97
			
PANC-1	Glu-Oct6	0	99
		300	98
		1000	97
	Phe-Oct6	0	99
		300	98
		1000	98

DU-145 and PANC-1 cells were incubated with 300 - 1000 nM peptides for 4 hr, then harvested, stained with FITC-annexin V/PI, washed and analyzed by flow cytometry to determine the cell viability using a FACScan flow cytometer and CellQuest Pro software. The average of 3 replicate experiments is shown.

### Uptake of all peptides was temperature-dependent, but uptake of peptides containing the Oct6 NLS only was inhibited by NaN_3_

**Figure 3:**
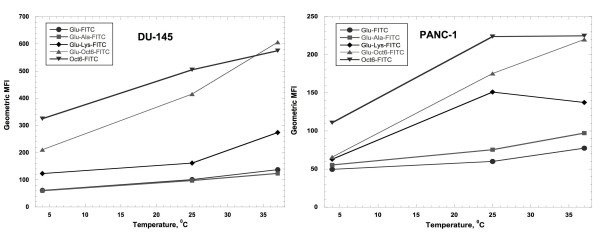
**Effect of temperature on peptide uptake**. Carboxyfluorescein-labeled peptides were incubated with DU-145 and PANC-1 cells at 300 nM over 4 hours at 4, 25, at 37°C. Harvested washed cells were analyzed on a FACScan flow cytometer. The results of representative experiments are shown.

In order to determine if peptides entered cells by diffusion or by an ATP-dependent process, the following experiments were performed. First, uptake of 300 nM peptides Glu-FITC, Glu-Ala-FITC, Glu-Lys-FITC, Glu-Oct6-FITC, and Oct6-FITC was compared at 4, 23, and 37°C for 4 hr in DU-145 cells. As shown in Figure 3, uptake of peptides tested was temperature-dependent, with best uptake observed at 37°C. Even at 4°C, peptides Glu-Oct6-FITC and Oct6-FITC exhibited greatly enhanced uptake, compared with the other peptides (average geometric MFIs were 486 for Glu-Oct6-FITC and 325 for Oct6-FITC, compared with average geometric MFI of 186 for Glu-Lys-FITC and geometric MFIs that were less than 100 for Glu-FITC and Glu-Ala-FITC). In the case of PANC-1 cells, a similar pattern of temperature dependence of uptake was observed. At 4°C, the average geometric MFIs for all peptides were found to be 50 to 110 (Figure 3); at 25°C the average geometric MFIs increased again increased slightly for Glu-FITC (to about 60) and Glu-Ala-FITC (to about 70). However, the average geometric MFI of Glu-Lys-FITC increased to about 150, that of Glu-Oct6 to about 175, and the average geometric MFI of Oct6-FITC was about 225. At 37°C, the average geometric MFIs of the Glu-FITC and Glu-Ala-FITC peptides were still less than 100 (about 70 and 80, respectively), whereas the average geometric MFI of the peptides containing the NLS either remained constant (Oct6-FITC), increased to over 200 (Glu-Oct6-FITC), or decreased (Glu-Lys-FITC). These data confirm the temperature-dependent uptake of Oct6 NLS-containing peptides in PANC-1 cells.

Usually, marked temperature dependence such as observed for these peptides is indicative of uptake by endoctytosis [15]. If this is the case, then uptake would be expected to be inhibited by depletion of ATP. To investigate this, experiments were performed for 4 hr at 37°C in the presence of NaN_3_. Table 3 shows that the addition of up to 1% NaN_3 _to DU-145 cells had little effect on the uptake of peptides Glu-FITC, Glu-Ala-FITC, or Glu-Lys-FITC at 300 nM (addition of 1% NaN_3 _inhibited the uptake of these peptides by less than 25%). However, the geometric MFI of 300 nM peptide Oct6-FITC was reduced from nearly 418 to 181 in the presence of 1% NaN_3_, while the geometric MFI of peptide Glu-Oct6-FITC was decreased from 151 to 114 in the presence of 1% NaN_3_. The addition of as little as 0.1% NaN_3 _inhibited the average geometric MFI for Oct6-FITC, reducing it from 418 to 293 (Table 3). These data indicate that uptake of the smaller peptides was ATP-independent but that uptake of the larger peptides containing the Oct6 NLS was ATP-dependent. Uptake of peptides lacking the Oct6 NLS was observed to be temperature-dependent but uptake was unaffected by up to 1% NaN_3_; on the other hand uptake of peptides Glu-Oct6-FITC and Oct6-FITC, both of which contain the Oct6 NLS, was inhibited by 1% NaN_3 _by over 50% (Tables 2 and 3). These preliminary studies demonstrated that uptake of peptides lacking the Oct6 NLS is likely through a non-endocytic mechanism and is not receptor-mediated whereas uptake of peptide containing the Oct6 NLS proceed through a different mechanism, but more studies have to be conducted to confirm if this observation is generalizable to other NLS peptides.

**Table 3:** Sodium Azide Inhibited Uptake of Peptides with Oct6 NLS

Peptide	**NaN**_**3**_**, %**	Avg Geometric MFI	% Inhibition
Glu-FITC	0	25.6	0
	0.1	26.4	0
	1.0	29.7	0
			
Glu-Ala-FITC	0	43.8	0
	0.1	56.9	0
	1.0	48.4	0
			
Glu-Lys-FITC	0	58.6	0
	0.1	61.8	0
	1.0	47.4	19.1
			
Glu-Oct6--FITC	0	151.4	0
	0.1	155.7	0
	1.0	114.4	24.8
			
Oct6-FITC	0	417.6	0
	0.1	293	29.8
	1.0	181	56.7

DU-145 cells were incubated with 300 nM peptides for 4 hr in the presence or absence of NaN_3_. Cells were washed and analyzed by flow cytometry to determine the geometric mean fluorescence intensities using a FACScan flow cytometer and CellQuest Pro software. The average of 3 replicate experiments is shown.

### Peptide Glu-Oct6-FITC colocalized to the nucleus

**Figure 4:**
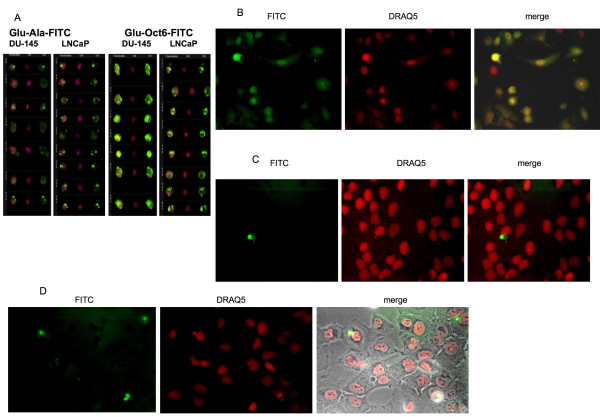
**Imaging studies**. **Figure 4A**: DU-145 and LNCaP cells were incubated with 250 nM Glu-Ala-FITC or Glu-Oct6-FITC for 4 hours, counterstained with 5 mM DRAQ 5, then fixed in paraformaldehyde and analyzed by imaging flow cytometry on an Amnis Imaging Cytometer. IDEAS software (Amnis) was used to generate and colorize the images. Across each row of images, one is viewing the same cell under different fluorescence excitations. Thus for each cell type and peptide, the left-most column shows the merged images, the middle column shows the DRAQ5 images, and the right-most column shows the FITC images (labeled below each set of 3 channels as **m **for merge, **D **for DRAQ5, and **F **for FITC). Analysis using IDEAS software revealed that Glu-Oct6-FITC distributed about 50% in nuclei and 50% in cytoplasm of cells, whereas only ~10% of nuclei were positive for DRAQ5 and FITC colocalization in the case of Glu-Ala-FITC. **Figure 4B: **Live cell imaging of Glu-Oct6-FITC by DU-145 cells. 500 nM Glu-Oct6-FITC was incubated with DU-145 cells overnight (necessary for imaging purposes). Cells were washed with phenol red-free buffer, counter-stained with DRAQ5 at 10 mM, and then examined under a Zeiss Axiovert 200 inverted phase contrast microscope with epifluorescence. The merged image confirms the presence of Glu-Oct6-FITC in nuclei. Merged images with and without Nomarski interference are shown. **Figure 4C and D: **DU-145 cells incubated with Glu-FITC and Oct6-FITC, as described for Figure 3B. Note the absence of FITC fluorescence in nuclei. Figure 3D merged image shows the Nomarski interference image overlayed on the fluorescence images.

Since flow cytometry only gives data regarding total shifts in fluorescence of a population, imaging flow cytometry and live imaging microscopy studies were performed to determine if the NLS augmented nuclear accumulation of the peptides. Imaging flow cytometry was chosen to provide data on intracellular distribution of FITC-labeled peptides. Inverted epifluorescence microscopy was chosen to eliminate inadvertant artifacts that may arise from fixation, such as nuclear translocation [16]. For these studies, the cell-permeable fluorochrome DRAQ5 was used because it readily enters and stains nuclei of viable cells [17]. Imaging flow cytometry revealed that peptide Glu-Oct6-FITC accumulated in the nucleus of DU-145 and LNCaP cells much better than did peptide Glu-Ala-FITC; at 250 nM Glu-Oct6-FITC was distributed approximately 50% in the nucleus and 50% in the cytoplasm. It was not retained in the plasma membrane (Figure 4A). In contrast, peptide Glu-Ala-FITC showed much less accumulation in the cells when the plasma membrane was excluded from the analysis. To be certain these results were not due to artifactual translocation due to fixation, live cell imaging studies were performed. We observed that 500 nM of peptide Glu-Oct6-FITC, but not peptides Glu-FITC, Glu-Ala-FITC, or Oct6-FITC, accumulated in the nuclei of DU-145 and LNCaP cells after 24 hrs incubation (Figure 4B-D). The translocation of peptides to nuclei was complete in the sense that FITC and DRAQ5 fluorescence colocalized in all viable cells, although cytoplasmic fluorescence was visible as well. The FITC distribution was somewhat punctate in the cytoplasmic portion of cells, leading us to conclude that the peptide may be accumulating within endosomes. These studies show that the presence of the glutamate-rich peptide EEEAA on the N-terminus of the Oct6 NLS of peptide Glu-Oct6-FITC enhanced nuclear colocalization as well as uptake.

### Peptide Glu-Oct6 facilitated transport of a synthetic oligonucleotide into prostatic and pancreatic cancer cell lines

**Figure 5:**
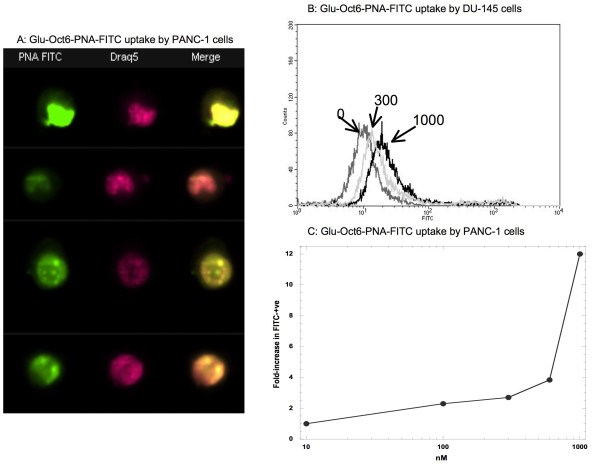
**Glu-Oct6 facilitated entry of a PNA into DU-145 and PANC-1 cells**. Glu-Oct6-PNA-FITC (0 to 1000 nM) was incubated with DU-145 or PANC-1 cells for 18 hr, at which time cells were harvested, washed, and processed for flow cytometry or imaging cytometry as described previously. **Figure A**: PANC-1 cells incubated with Glu-Oct6-PNA-FITC. IDEAS software (Amnis) was used to generate and colorize the images. Across each row of images, one is viewing the same cell under different fluorescence excitations. Thus for each cell type and peptide, the right-most column shows the merged images, the middle column shows the DRAQ5 images, and the left-most column shows the FITC images. Four thousand cells total were analyzed. **Figure B**: Uptake of Glu-Oct6-PNA-FITC by DU-145 cells quantified by flow cytometry. **Figure C**: Concentration-dependent increase in FITC fluorescence by PANC-1 cells incubated with Glu-Oct6-PNA-FITC, assessed by flow cytometry.

Because the ultimate goal of these studies is to use peptide Glu-Oct6 to ferry in a therapeutic cargo, the hybrid molecule Glu-Oct6-PNA was synthesized, which consisted of Glu-Oct6 at the N terminus and the carboxyfluorescein peptide nucleic acid (PNA) having the inert sequence TATGATCTCCTCCGT-lysyl-FITC at the C terminus (Glu-Oct6-PNA-FITC). Glu-Oct6-PNA-FITC was incubated with DU-145 and PANC-1 cells at 0, 300, and 1000 nM for 18 hr, at which time cells were harvested and processed for flow cytometry. Figure 5 shows that increasing the concentration of Glu-Oct6-PNA-FITC increased the MFI of the cells, and the percent of cells positive for FITC fluorescence. Analysis of PANC-1 cells by imaging flow cytometry, which can quantify colocalization of fluorescent dyes to subcellular organelles [18] revealed that Glu-Oct6-PNA-FITC colocalized to a great extent with the nuclear stain DRAQ5 (Figure 5A). IDEAS software analysis of the 4 to 5 thousand cellular events acquired revealed that the extent of colocalization was approximately 70% nucleus and 20% cytoplasmic across all events examined (Table 4). The fluorescent intensities for PNA-FITC in nuclei of both cell lines was much greater than those measured in the cytoplasmic portions (Table 4), indicating that the PNA accumulated in the nuclei to a much greater extent than in the cytoplasm. The percents do not add up to 100% because they exclude the portion that localized to the cell membrane asa well as whatever amount of PNA clung to cellular debris. In addition, a small but highly fluorescent population of cells (having a MFI greater than 10^3^) was apparent, even as low as 300 nM (Figure 5B). Furthermore, PANC-1 cells increased in proportion of FITC positive with increasing concentration of Glu-Oct6-PNA-FITC (Figure 5C). Therefore, conjugating Glu-Oct6 peptide to a PNA facilitated entry and nuclear colocalization of the PNA into both DU-145 and PANC-1 cell lines.

**Table 4:** Glu-Oct6 Facilitated Nuclear Colocalization of PNA 13778a

Cell line	mean % nuclear FITC	mean nuclear FITC intensity	mean % cyto. FITC	mean cyto. FITC intensity
DU-145	68.04	274,597	22.48	76,943
PANC-1	72.61	322,794	21.8	68,465

Cells were incubated with 1000 nM Glu-Oct6-PNA-FITC for 18 hr, at which time cells were fixed with 4% paraformaldehyde, stained with 5 μM DRAQ5 for nuclear localization, and analyzed on an Amnis ImageStream imaging cytometer.

Four to 5 thousand events were collected and analyzed using IDEAS software (Amnis).

### Peptide Glu-Oct6 facilitated transport of an anti-STAT3 PNA into cancer cell lines, resulting in apoptosis

To determine if the creation of an anti-STAT3 PNA that is transported into cells by the novel CPP Glu-Oct6 is feasible, a PNA was synthesized bearing Glu-Oct6 on the N terminal portion, plus an anti-STAT3 sequence, 13410a [2]. A control PNA consisted of the 13410a sequence without a peptide coupled to it. These were incubated with DU-145 or PANC-1 cells for48hr, at which times cells were harvested, stained, and analyzed by flow cytometry to quantify apoptosis. Table 5 shows that significant apoptosis was induced in both DU-145 and PANC-1 cells by as little as 600 nM Glu-Oct6-13410a (48 and 69% respectively; p < 0.01 by repeated ANOVA; Table 5), whereas addition of PNA 13410a, lacking any CPP, induced very little apoptosis at 1000 nM (1% or less; Table 5). These data are encouraging for the future development of experimental therapeutics using Glu peptides bound to transcription factor NLS peptides for aiding the translocation of active payloads into cells. Further work in this area of peptide design is planned.

**Table 5:** PNA Glu-Oct6-13410a Induced Apoptosis in DU-145 and PANC-1 Cells

DU-145					
**nM PNA**	**0**	**300**	**600**	**1000**	**13410a**

% apop.	0.05	8.7	48.7*	82.5*	2.9
SD	0.04	7.7	24.1	5.9	4.6

**PANC-1**					

**nM PNA**	**0**	**300**	**600**	**1000**	**13410a**

% apop.	0.3	17.4	69.6*	84.6*	1.1
SD	0.2	14.6	5.9	14.6	0.8

Cells were incubated with PNAs Glu-Oct6-13410a or 13410a for 48 hr, at which time they were harvested and stained with FITC-annexin V plus PI, then apoptosis was quantified by flow cytometry. PNA 13410a was added at 1000 nM. The percents apoptosis are the means of 3 experiments; SD = standard deviation.

* p < 0.01 by repeated ANOVA

## Discussion

The use of NLS peptides was explored because of their potential to ferry therapeutic cargoes efficiently. Previously, it was observed that the Oct6 NLS peptide accumulated in the endosomal compartments of cells [13]. However, it was observed that addition of peptide EEEAA to the N-terminus of the Oct6 NLS enhanced cellular uptake and also enhanced nuclear localization. The AA residues were added so that the Glu peptide would be spatially separated from the NLS portion, to maximize binding of each. Ala-rich linker peptides have been described before to link functional domains of various proteins [19]. It was further observed that although Glu-Oct6 facilitated entry of a PNA into DU-145 cells, higher concentrations and longer incubation times were required for the PNA than for the peptide Glu-Oct6-FITC. It is conceivable that there is a large energy barrier to overcome for efficient transport of PNA into cells, despite their neutral charge and despite the apparently enhanced cell uptake properties of Glu-Oct6 ([20] and Figures). It is entirely possible that Glu-Oct6 would function as a more efficient CPP if the form of the therapeutic payload were changed from a PNA to a different entity, such as a locked nucleic acid. Notwithstanding, Glu-Oct6 deserves further study as a potential probe for studying nuclear localization events, and as a CPP for ferrying other forms of therapeutic payloads, such as peptides or liposomes.

Temperature-dependence of peptide uptake has been found to correlate as well with segregation to intracellular compartments. Fretz and coworkers observed that at lower temperatures (4 to 12°C), L- and D-octa-arginine peptides partitioned across nuclear and cytoplasmic compartments equally, moving to the endosomes of CD34^+ ^leukemia cells when ambient temperature rose to 30°C and higher [21]. They further observed that raising concentration affected which intracellular compartments were labeled by peptides [21]. Similarly, temperature-dependent uptake of peptide Glu-Oct6-FITC (Figure 3) was observed in DU-145 and PANC-1 cell lines; furthermore Glu-Oct6-FITC partitioned across nuclei and cytoplasm (Figure 4A and 4B), whereas neither peptides Glu-FITC nor Oct6-FITC exhibited appreciable nuclear localization (Figure 4C and 4D). The Oct6 transcription factor is known to shuttle between cytoplasm and nucleus [22]; this would be expected of transcription factors. However, the NLS peptide of a transcription would not be expected to shuttle, since it is not activated. Rather, one might expect that an NLS peptide accumulates in one or more select organelles. Indeed, Ragin and coworkers observed such accumulation with regard to the Oct6 NLS and others [13]; accumulation across cytoplasm and nucleus by Glu-Oct6-FITC indicates that addition of the Glu-rich peptide to the Oct6 NLS prevents the endoplasmic sequestration by a mechanism that has yet to be explored. Several mechanisms of peptide uptake are known; ATP-dependent and ATP-independent peptide uptake are two major differentiating features of peptide uptake but by no means the only ones. Even within the same cell line, investigators have observed multiple modes of peptide uptake; endocytic and non-endocytic modes of uptake were noted in the V79 and PC12 cell lines [23]. Furthermore, the uptake of NLS peptides by the MCF-7 breast cancer cell line was found to be temperature-dependent but unaffected by the presence of NaN_3 _[13].

PNAs have been a focus of cancer researchers for over a decade. Early work on PNAs for cancer therapy showed that anti-sense PNAs directed against the androgen receptor and TATA-binding protein genes worked by hybridization with CAG triplet repeats in LNCaP and DU-145 cell lines; no binding to *c-myc*, which lacks the CAG repeats was observed [24]. Although marked effects on the transcriptosomes of the androgen receptor and TATA-binding proteins genes were observed, the PNAs were not designed to be therapeutically active. Elegant studies on T47D and MCF-7 breast cancer cell lines with PNA-peptide conjugates targeting human progesterone receptor gene isoforms A and B revealed the extent to which expression of progesterone receptor protein was attenuated [25]. More recently, investigators showed therapeutic efficacy in a mouse model of Burkitt's lymphoma using an anti-sense PNA targeted to the Em enhancer region of the H chain locus, which in Burkitt's lymphoma is transposed near the *c-myc *locus [26].

Glutamate receptors are known to be overexpressed by cancer cells. In prostate cancer, the best known is PSMA, which binds carboxy glutamates [27,28]. PSMA we believe is not involved because DU-145 cells are PSMA-negative [29], and because the glutamate is on N-termini of the peptides. Metabotropic glutamate receptors are usually found on neuronal cells but are found to be aberrantly expressed by malignant cells. These glutamate receptors mediated 5-fluorouracil resistance in human colon cancer cells [30]. Glutamate receptors are implicated in transformation to malignancy; it's hypothesized that glutamate receptors overexpression may be a common feature of tumor pathogenesis. Thus glutamate receptors may be typical of what Glu-Oct6 binds to on DU-145, LNCaP, and PANC-1 cells. The activity of normal glutamate receptors in ectopic cellular environments may involve signaling pathways, which dysregulate cell growth, ultimately leading to tumorigenesis. Thus, dysregulated and aberrantly-expressed glutamate receptors may function as oncogenes [31]. Malignant prostatic neuroendocrine cells proliferate more when glutamate receptors are stimulated; they use glutamate as a substrate for NADH biosynthesis, producing increased levels of free fatty acids. These activities correlate with the aggressive nature of these tumors [32]. Glutamate receptors have been understudied and certainly have not yet been widely used for cancer-specific targeting. Since glutamate receptors are overexpressed on a variety of solid tumors [31], they should lend themselves well to cancer cell targeting by a variety of strategies, including CPP design. This should be the focus of future work.

Recent papers on peptide uptake indicates that nuclear colocalization, even by transcription factor NLS peptides, is not easily achieved [13]. Here we describe the enhancement of uptake and nuclear localization of a NLS through addition of a peptide. Whether addition of the peptide enhances uptake and nuclear localization when coupled to the NLS of other transcription factors and whether the enhancement is sequence-specific is under investigation.

## Conclusions

The use of the Oct6 NLS peptide as a CPP was explored. Peptide Glu-Oct6-FITC was shown to gain entry into DU-145, PANC-1, and LNCaP cells quickly and efficiently, and localized to the nucleus. Its ability to function as a CPP was concentration- and temperature-dependent, and abrogated in the presence of azide. The homologous peptide Oct6-FITC, which consisted of the Oct6 NLS peptide alone and lacked the N-terminal glutamate-rich peptide, did not localize to the nucleus. The ability of Glu-Oct6 to function as a CPP was lost when Phe, Tyr, or Asn were substituted for the Glu residues. Peptide Glu-Oct6 facilitated entry of a carboxylysyl-fluorescein PNA into DU-145 and PANC-1 cells. Finally, peptide Glu-Oct6 facilitated transport of an anti-STAT3 PNA into DU-145 and PANC-1 cells, resulting in significant apoptosis. Therefore, Glu-Oct6 may be a peptide useful for therapeutic applications.

## Methods

### Synthesis of Peptides and Peptide-PNA

Peptides used are listed in Table 1 and are referred to by their synthesis numbers for convenience. The carboxyfluorescein and carboxydansyl amino acids were purchased from Bachem. All peptides used had a molar ratio of FITC or dansyl to peptide of 1. All peptides were synthesized by the Molecular Resources Facility at the University of Medicine and Dentistry, New Jersey Medical School (Newark, NJ) on an Applied Biosystems model 433 peptide synthesizer using *FMoc *chemistry. After cleavage and deprotection, the peptides were purified by high-performance liquid chromatography (HPLC) and analyzed by both HPLC and sequencing on an Applied Biosystems Procise 494C sequenator. The FITC-labeled peptide-PNAs FITC-PNA-EEE, FITC-TAT-PNA, and Glu-Oct6-PNA-FITC were synthesized by BioSynthesis (Lewisville, TX). They were purified by HPLC and their structures verified by MALDI-TOF. Because of the requirements of PNA chemistry, FITC was added to the C-terminus of Glu-Oct6-PNA as lysyl FITC.

### Cells

DU-145 and LNCaP cells were the gift of Dr. James Turkson (University of Central Florida, Orlando, FL). DU-145 cells were grown in DMEM/Ham's F12 (Invitrogen, Carlsbad, CA) plus 10% newborn bovine serum (Hyclone, Logan, UT). LNCaP cells were maintained in RPMI-1640 (Invitrogen) plus fetal bovine serum (Hyclone). PANC-1 cells were the gift of Dr. James Freeman, University of Texas Health Sciences Center, San Antonio TX. They were grown in the same medium as the DU-145 cells. Cell viabilities were determined using fluorescein diacetate (Sigma Chemical Co., St. Louis, MO) and a Universal RIII fluorescence microscope (Zeiss, Jena, Germany).

### Uptake/Fluorescence Quantification and Nuclear Colocalization Studies

Peptides were added to subconfluent cultures of cells at times, temperatures, and concentrations indicated in experiments. Concentrations ranging from 0 to 1000 nM were assayed. Fluorescence was normalized using calibration beads (Becton-Dickinson; BD). Cells were analyzed in the presence of 5 mM 7-AAD (eBioscience, San Digeo CA) to gate on live events. Fluorescence was quantified, after cells were harvested, using a BD FACScan flow cytometer. At least 10,000 events were acquired using CellQuest Pro software and an Apple Macintosh G4 dual coprocessor computer running OS X 10.3.9. Fluorescence detectors on the instrument were standardized prior to each acquisition run, so that fluorescence intensities from different days could be compared. Because the FACScan employs logarithmic amplifiers on the fluorescence detectors, the more accurate parameter with which to compare fluorescence intensities for different samples is the geometric mean fluorescence intensity (geometric MFI). For the studies examining uptake of carboxyfluorescein peptides plus carboxydansyl peptides, fluorescence was quantified on a BD LSR II flow cytometer. In nuclear colocalization studies, cells were stained with the nuclear stain DRAQ5 (Axxora, San Diego, CA) at 5 mM final concentration following uptake of peptides. Fixed cells (4% paraformaldehyde/DPBS) were then analyzed on an Amnis ImageStream 200 imaging cytometer using IDEAS 3.0 software. Four to five thousand events were collected for analysis.

### Live Cell Imaging Studies

A Zeiss Axiovert 200 inverted phase-contrast microscope outfitted with epifluorescence was used for live imaging studies. Subconfluent cultures of cells in 12-well plates were incubated with 500 nM peptides as indicated. DRAQ5 (Axxora; 10 mM final concentration) was added for the last hour of incubation, then the cells were washed twice with warm phenol red-free buffer. Cells were examined in phenol red-free buffer plus 10% serum. Images were acquired and analyzed using Zeiss Axiovision software.

### Apoptosis Studies

DU-145 and PANC-1 cells were incubated with PNAs Glu-Oct6-13410a or 13410a (no CPP attached) at 0, 300, 600, or 1000 nM for 48 hr. Cells were harvested, then stained with FITC-annexin V (Abcam; Cambridge, MA) and counterstained with propidium iodide (Sigma, St.Louis, MO). Cells were analyzed on a BD FACScan for fluorescence in the FL1 and FL3 channels; CellQuest Pro software was used to quantify fluorescence and determine the extent of apoptosis.

### Statistical Analysis

The graphing program Kaleidagraph 4.2 (Synergy Software, Reading, PA) and the statistical program InStat3 (GraphPad Software, San Diego, CA) were used for data analyses unless otherwise indicated.

## Competing interests

The authors declare that they have no competing interests.

## Authors' contributions

HDL, KG, SR, and AH performed the peptide and peptide-PNA studies. RJD synthesized and purified the carboxyfluorescein and carboxydansyl peptides. DB performed the microscopy studies and analysis. AS suggest the Tyr-Oct6 modification of the peptide. BEB designed the remaining peptides and experiments, performed the flow cytometry aquisition and analyses, and the statistical analyses. All authors read and approved the final manuscript.
